# Enhanced Bacterial Fitness Under Residual Fluoroquinolone Concentrations Is Associated With Increased Gene Expression in Wastewater-Derived *qnr* Plasmid-Harboring Strains

**DOI:** 10.3389/fmicb.2018.01176

**Published:** 2018-06-08

**Authors:** Ella Kaplan, Roberto B. M. Marano, Edouard Jurkevitch, Eddie Cytryn

**Affiliations:** ^1^Department of Soil Chemistry, Plant Nutrition and Microbiology, Institute of Soil, Water and Environmental Sciences, Volcani Center, Agricultural Research Organization, Beit Dagan, Israel; ^2^Department of Agroecology and Plant Health, The Robert H. Smith Faculty of Agriculture, Food and Environment, The Hebrew University of Jerusalem, Rehovot, Israel

**Keywords:** *qnr* genes, wastewater treatment, plasmid, qPCR expression analysis, fitness

## Abstract

Plasmids harboring *qnr* genes confer resistance to low fluoroquinolone concentrations. These genes are of significant clinical, evolutionary and environmental importance, since they are widely distributed in a diverse array of natural and clinical environments. We previously extracted and sequenced a large (∼185 Kbp) *qnrB*-harboring plasmid, and several small (∼8 Kbp) *qnrS*-harboring plasmids, from *Klebsiella pneumoniae* isolates from municipal wastewater biosolids, and hypothesized that these plasmids provide host bacteria a selective advantage in wastewater treatment plants (WWTPs) that often contain residual concentrations of fluoroquinolones. The objectives of this study were therefore to determine the effect of residual fluoroquinolone concentrations on the growth kinetics of *qnr* plasmid-harboring bacteria; and on the copy number of *qnr* plasmids and expression of *qnr* genes. Electrotransformants harboring either one of the two types of plasmids could grow at ciprofloxacin concentrations exceeding 0.5 μg ml^-1^, but growth was significantly decreased at concentrations higher than 0.1 μg ml^-1^. In contrast, plasmid-free strains failed to grow even at 0.05 μg ml^-1^. No differences were observed in plasmid copy number under the tested ciprofloxacin concentrations, but *qnr* expression increased incrementally from 0 to 0.4 μg ml^-1^, suggesting that the transcription of this gene is regulated by antibiotic concentration. This study reveals that wastewater-derived *qnr* plasmids confer a selective advantage in the presence of residual fluoroquinolone concentrations and provides a mechanistic explanation for this phenomenon.

## Introduction

The extensive use, and misuse of antibiotics in the past half century has significantly contributed to the proliferation of antibiotic resistant bacteria (ARB), and associated antibiotic resistance genes (ARGs) that confer resistance to many of the clinically prescribed antibiotic compounds (Levy and Marshall, 2004; Schmieder and Edwards, 2012). Although antibiotic resistance has traditionally been associated with clinical environments, there is substantial evidence suggesting that anthropogenically-impacted hotspots such as animal husbandry facilities and wastewater treatment plants (WWTPs) can contribute to antibiotic resistance in natural environments and thereby impact the global scope of antibiotic resistance (Davies and Davies, 2010; Allen, 2014; Gatica et al., 2016). Many ARGs are carried on mobile genetic elements (MGEs) such as IS-sequences, integrons and plasmids. This facilitates the horizontal transfer of ARGs within and between environmental microbiomes, thus playing a central role as one of the most powerful forces in microbial evolution and ecology (Frost et al., 2005).

Fluoroquinolones bind to the holoenzyme of bacterial gyrase/topoisomerase IV and double-stranded nicked DNA in the bacterial chromosome replication fork, thus preventing its progress, leading to cell cycle arrest and eventually to cell death (Dalhoff, 2012). Due to their chemical characterization, fluoroquinolones accumulate in dewatered sludge, where concentrations of up to 50 mg/kg dry weight have been detected (Golet et al., 2002). Resistance to fluoroquinolones is traditionally associated with specific mutations in chromosomal genes encoding for gyrase/topoisomerase enzymes. However, plasmid-mediated quinolone resistance (*qnr*) genes confer resistance to sub-clinical levels of fluoroquinolones that are approximately one order of magnitude lower than minimum inhibitory concentration (MIC) levels associated with chromosomal mutations (Martínez-Martínez et al., 1998; Robicsek et al., 2006). This occurs by competitive binding of the Qnr proteins to the gyrase, prior to its binding to the nicked DNA, consequently resulting in declining of assembly of the antibiotic recognition site. (Martínez-Martínez et al., 1998; Robicsek et al., 2006; Strahilevitz et al., 2009). The binding of Qnr to the bacterial gyrase partially interferes with the replication fork progression and therefore, notwithstanding the fact that it keeps the cell from succumbing to the lethal effect of the fluoroquinolones, Qnr binding to the gyrase slow replication fork progression, leading to reduction of the growth rate of the bacterial culture (Tran et al., 2005). Although previous studies have suggested that *qnr* genes originated in natural environments (Poirel et al., 2005), they have evolved in a wide array of commensal and pathogenic bacteria and are strongly associated with multidrug resistance (Perry and Wright, 2013; Marti et al., 2014).

Albeit lower than the MIC conferred by genomic mutations, Qnr defense should not be easily disregarded, since sub-inhibitory ciprofloxacin concentrations are common in anthropogenic environments such as sewage and treated wastewater; and because the *qnr* genes are mainly carried on transferrable plasmids, making them of high ecological and evolutionary importance since they can be disseminated from anthropogenic sources into microbiomes in natural environments (Marti et al., 2014). Furthermore, plasmid-mediated quinolone resistance may allow the “window of opportunity” needed for the development of point mutations in the gyrase, leading to resistance to clinically relevant fluoroquinolone concentrations (Drlica, 2003; Cantón and Morosini, 2011; Marti et al., 2016). Indeed, the relevance of *qnr*s in the environmental resistome can also be demonstrated by the fact that *qnrS*, for instance, although sometimes rarely detected in the clinical settings, is very prevalent in WWTPs and has a very broad host range suggesting a competitive advantage of these plasmids in in this environment (Kaplan et al., 2015; Marti et al., 2016).

In a previously published study, we isolated numerous ciprofloxacin-resistant *Klebsiella* strains from dewatered biosolids of a large municipal wastewater treatment facility, and extracted, transformed, characterized, and fully sequenced seven *qnr*-bearing plasmids (Kaplan et al., 2013). The first was a mega-plasmid of 185 Kbp that encoded 10 different ARGs, including *qnrB*. This plasmid shared a high level of sequence identity with a wide range of previously described, clinically associated pKP3-like plasmids. The other six plasmids were much smaller (∼8 Kbp) and were highly similar (>95% identity) to pGNB2, a plasmid previously detected in a German wastewater treatment facility by plasmid capture (Bönemann et al., 2006) and pBRST7 from an *Aeromonas hydrophila* strain isolated from diseased fish in an Indian aquaculture system (Majumdar et al., 2011).

It is traditionally believed that maintaining plasmids within host cells confers significant fitness costs due to the need to synthesize extra nucleotides and enzymes for replication and transfer; and therefore, the maintenance of a plasmid within a cell requires a selective advantage that exceeds the abovementioned fitness cost (Martínez and Rojo, 2011; Hernando-Amado et al., 2017). While most studies have evaluated the cost/benefit of harboring plasmids under minimal inhibitory antibiotic concentrations, studies by Gullberg et al. (2011) demonstrated that several resistance mechanism conferred significant selective advantages at sub-inhibitory concentration of antibiotics. The realization that environmentally relevant antibiotic concentrations can select for ARGs has tremendous ramifications for both the spread and evolution of antibiotic resistance (Gullberg et al., 2011; Cytryn, 2013).

The aim of this study was to assess the impact of selective pressure in the form of sub-therapeutic and clinical fluoroquinolone concentrations, on the growth dynamics, plasmid abundance and *qnr* gene expression in bacteria harboring *qnr* plasmids that were isolated from municipal WWTPs. The growth of naïve and electro-transformed *Escherichia coli* DH10B strains with the above-described *qnrB-* and *qnrS*-harboring plasmids was monitored in the presence of different concentrations of ciprofloxacin; in tandem, plasmid abundance and *qnrS* gene expression were quantified using real-time PCR.

## Materials and Methods

### Growth Rates of *E. coli* DH10B Electrotransformants Under Different Ciprofloxacin Concentrations

*Escherichia coli* DH10B competent cells (20 μl) electrotransformed with either the 8 Kbp *qnrS*-harboring plasmid pKPSH213.55, or the 185 Kbp *qnrB*-harboring plasmid pKPSH11-XL (Kaplan et al., 2015), were grown in 180 μl LB-broth with the following concentrations of ciprofloxacin: 0, 0.05, 0.1, 0.2, 0.3, 0.4, 0.5, 1.0, 2.0, and 4.0 μg ml^-1^. In tandem, plasmid-free DH10B cells were grown under the same ciprofloxacin concentrations as a control. Cultures were grown in sterile 96-well microwell plates (Thermo Fisher Scientific Inc., Denmark) following an initial inoculation of 2 × 10^4^ cells ml^-1^ in an automated Spark 10 M multimode microplate reader (Tecan, Zurich, Switzerland) at 37°C, gently shaking every 20 min; and cell density was closely measured every 40 min for at least 30 h.

In addition to microplate experiments, naïve DH10B cells and pKPSH213.55-harboring DH10B strain were also grown in 5 ml of sterile LB-broth at 37°C under constant shaking to assess growth dynamics in large volumes, and to extract nucleic acids used for the *qnrS* abundance and expression experiments described below. In these experiments, four different ciprofloxacin concentrations: 0, 0.1, 0.4, and 1.0 μg ml^-1^ were evaluated. The initial cell density was 6 × 10^4^ cells ml^-1^, in three biological repeats, and growth was monitored periodically using a biochrom WPA spectrophotometer (Biochrom, Ltd., Cambridge, England). Cultures were harvested at mid-log phase, at 2 × 10^6^ cells ml^-1^ and immediately frozen on liquid N_2_. Naïve DH10B cells grown without antibiotic and DH10B pKPSH213.55 cells grown with 0 and 0.1 μg ml^-1^ ciprofloxacin were harvested after 2.5 h and DH10B pKPSH213.55 cells with 0.4 μg ml^-1^ ciprofloxacin were harvested after 6 h. DH10B pKPSH213.55 cells grown with 1.0 μg ml^-1^ began collapsing were harvested after 7 h, at 5 × 10^5^ cells ml^-1^. Due to the low density of cells in this culture, duplicate cell cultures were used for each nucleic acid extraction.

### Nucleic Acid Extraction, Purification and Synthesis of Single Strand cDNA From Mid Log-Phase Electrotransformants

DNA and RNA from the harvested cell cultures were extracted using the Exgene Cell SV kit (GeneAll, Seoul, South Korea) and the EZ-RNA kit (Biological Industries, Beit HaEmek, Israel), respectively; according to the manufacturers’ instructions. Residual DNA was removed from the RNA samples by digesting with DNase I for 20 min at room temperature (Sigma, St. Louis, MO, United States). Synthesis of single strand cDNA was achieved using ImProm-II^TM^ Reverse-Transcriptase (Promega, Madison, WI, United States) with random primers and 1.0 μg of total RNA template.

RNA, cDNA, and genomic DNA concentrations were measured with a Qubit^TM^ 3.0 Fluorometer (Thermo Fisher Scientific, United States) using reagents and protocols supplied by the manufacturer.

### Quantitative PCR Assessment of Plasmid Copy Number and *qnrS* Gene Expression Levels

Plasmid copy number (abundance of *qnrS*) and *qnrS* gene expression levels in the naïve and pKPSH213.55-transformed DH10B cells grown in the presence of 0, 0.1, 0.4, and 1.0 μg ml^-1^ were monitored by real-time quantitative PCR (qPCR). Triplicates from whole genomic DNA and cDNA for each of the four ciprofloxacin concentrations were diluted 10-fold and 1 μl was used in a 20 μl final reaction volume together with 0.5 μM of each primer and 1X SYBR Green Master Mix. For *qnrS*, the SYBR^®^ DyNAmo Flash kit (Thermo Scientific^TM^) was used together with the primers *qnrS*rt-F11 (5^′^-GACGTGCTAACTTGCGTGAT-3^′^) and *qnrS*rt-R11 (5^′^-TGGCATTGTTGGAAACTTG-3^′^; Marti and Balcázar, 2013) to generate a 118 bp amplicon. In tandem, total bacterial abundance was estimated by targeting the 16S rRNA gene using the universal primers 331-F (5^′^-TCCTACGGGAGGCAGCAGT-3^′^) and 518-R (5^′^-ATTACCGCGGCTGCTGG-3^′^) (Nadkarni et al., 2002; Lopez et al., 2003). Samples were denatured at 95°C for 7 min, followed by 40 cycles at 95°C for 10 sec and 60°C for 30 sec. Three technical replicates were conducted for each individual sample. Efficiency of reactions was monitored in each run by means of an in internal standard curve using a 10-fold dilution of standards ranging from 10^7^ to 10^2^ copies per reaction, done in duplicates. Reported efficiency was between 96 and 98.6% for all runs, and *R*^2^-values were greater than 0.99. For both qPCR primer sets, the template for the standard curve used the pNORM1 plasmid (courtesy of C. Merlin). This plasmid is a standard pEX-A vector containing a synthetic sequence combining fragments that cover both of the target gene amplicons (Rocha et al., 2018). Additionally, presence of qPCR inhibitors in samples was assessed using an additional 100-fold dilution as previously suggested (Bustin et al., 2009). All runs were performed using a StepOnePlus real-time PCR system (Applied Biosystems, Foster City, CA, United States) and data analysis was conducted using the StepOne software v2.3 (Applied Biosystems, Foster City, CA, United States).

Genomic DNA was targeted to estimate plasmid copy number per cell. Specifically, *qnrS* values were divided by 16S rRNA values and multiplied by seven, which is the documented number of 16S rRNA copies in the *E. coli* DH10B genome (Durfee et al., 2008). For *qnrS* gene expression analyses, the estimated *qnrS* copy number per sample was determined by normalizing to both *E. coli* DH10B abundance and to cDNA concentration. Briefly, for cell normalization, average copy number values from qPCR technical replicates were multiplied by the dilution factor used in qPCR and the final volume in μl of the cDNA sample after reverse-transcription, and then divided by the estimated cell number at harvest used for RNA extraction. For cDNA normalization, copy number from each qPCR technical replicate was multiplied by the dilution factor used in qPCR and divided by the concentration expressed in ng μl^-1^ of the cDNA sample.

To determine the statistical significance of ciprofloxacin concentration on both plasmid copy number and *qnrS* gene expression, we applied unpaired *t*-tests, comparing samples grown in the presence of different ciprofloxacin concentrations to the control grown without ciprofloxacin (*t*-test was performed using GraphPad Prism version 6.00 for Windows, GraphPad Software, La Jolla, CA, United States).

## Results

### Impact of Ciprofloxacin Concentration on Growth Dynamics of *qnrS-* and *qnrB-*Harboring Plasmids

**FIGURE 1:**
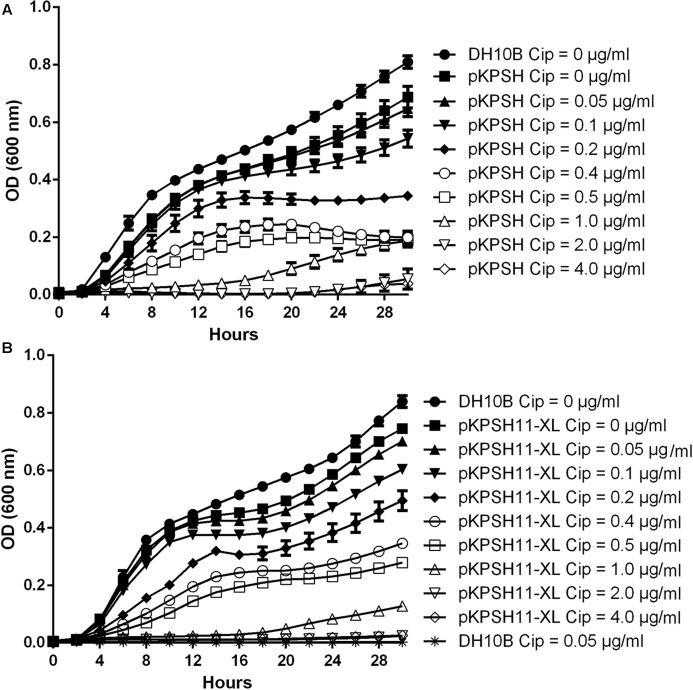
Growth dynamics of *qnrS*- and *qnrB*-encoding plasmids in DH10B *Escherichia coli* electrotransformants exposed to different ciprofloxacin concentrations. Naïve and pKPSH213.55 (*qnrS*)-transformed cells **(A)**. Naïve and pKPSH11-XL (*qnrB*)- transformed cells **(B)**. Plasmid-free DH10B cells did not grow under any of the indicated ciprofloxacin concentrations. Cip - ciprofloxacin.

Growth dynamics of naïve *E. coli* DH10B and *E. coli* DH10B cells electrotransformed with the wastewater-derived plasmids pKPSH213.55 (8 Kbp *qnrS*-harboring plasmid) and pKPSH11-XL (185 Kbp *qnr*-harboring plasmid), are shown in **Figure 1**. Naïve untransformed *E. coli* DH10B cells did not grow in the presence of the lowest ciprofloxacin concentration measured (0.05 μg ml^-1^). In contrast, *qnr* plasmid-harboring strains grew at concentrations of up to 0.5 μg ml^-1^, albeit with incrementally decreasing growth rates and lower maximum cell densities at ciprofloxacin concentration above 0.1 μg ml^-1^ (**Figure 1**).

Collectively, growth kinetics of both transformants could grossly be divided into three groups: (A) at ciprofloxacin concentrations below 0.1 μg ml^-1^, cells exhibited short lag phases (∼2 and ∼4 h for *qnrS-* and *qnrB*-harboring cells, respectively), high growth rates and reached high densities. (B) at ciprofloxacin concentrations that ranged from 0.2 to 0.5 μg ml^-1^, cells exhibited longer lag phases (∼4 h), lower growth rates and reached lower cell densities. (C) at ciprofloxacin concentrations that exceeded 1 μg ml^-1^, cells exhibited extremely long lag phases (∼12 and ∼20 h for *qnrS-* and *qnrB*-harboring cells, respectively, under 1 μg ml^-1^ ciprofloxacin and more than 24–30 h for 2 and 4 μg ml^-1^, respectively), very slow growth rates and reached low cell densities.

Despite the fact that the *qnrB*-harboring plasmid pKPSH11-XL is more than 20 times larger than the *qnrS*-harboring plasmid pKPSH213.55, we did not observe significant differences in the growth kinetics of the two transformants, suggesting that the fitness cost of the increase plasmid size is negligible (**Supplementary Figure S1**).

### Impact of Ciprofloxacin Concentration on *qnrS*-Harboring Plasmid Copy Number

**FIGURE 2:**
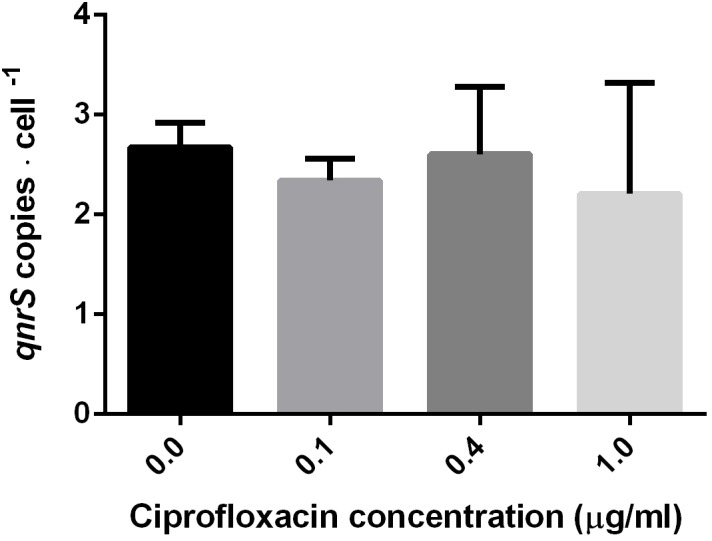
Estimated copy number of pKPSH213.55 plasmids per bacteria purified from cultures at mid-log phase cells grown in media containing different ciprofloxacin concentrations (0, 0.1, 0.4, and 1.0 μg ml^-1^). Plasmid copy number was inferred by normalizing qPCR-based *qnrS* copy number to the seven gene copies of 16S rRNA in DH10B. Cells grown in 0 and 0.1 μg ml^-1^ ciprofloxacin were harvested after 2.5 h; cells grown in 0.4 μg ml^-1^ ciprofloxacin were harvested after 6 h; and cells grown in 1.0 μg ml^-1^ ciprofloxacin were harvested after 7 h.

To examine the short-term impact of ciprofloxacin exposure (2.5–7 h) on *qnr* plasmid copy number, DNA was extracted from mid-log phase pKPSH213.55-harboring *E. coli* DH10B electrotransformants grown in 0, 0.1, 0.4, and 1.0 μg ml^-1^ ciprofloxacin. The *qnrS* and 16S rRNA gene copy numbers were determined by qPCR, and pKPSH213.55 abundance was estimated by *qnrS* to 16S rRNA copy number ratios. *E. coli* DH10B strains harbor seven copies of 16S rRNA, and therefore, we estimated that each cell maintained approximately 2–3 copies of pKPSH213.55 per cell. The three ciprofloxacin concentrations tested had no significant impact on plasmid copy number relative to the bacteria that were grown without antibiotic (**Figure 2**).

### Impact of Ciprofloxacin Concentration on *qnrS* Expression

**FIGURE 3:**
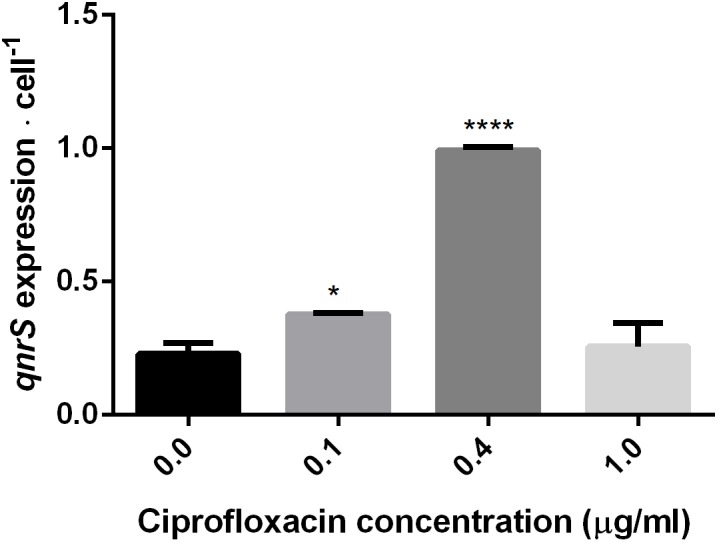
Quantification of *qnrS* transcripts normalized to estimated bacterial cell number at mid-log phase under different ciprofloxacin concentrations (0, 0.1, 0.4, and 1.0 μg ml^-1^). Cells grown in 0 and 0.1 μg ml^-1^ ciprofloxacin were harvested after 2.5 h; cells grown in 0.4 μg ml^-1^ ciprofloxacin were harvested after 6 h; and cells grown in 1.0 μg ml^-1^ ciprofloxacin were harvested after 7 h. The reported significance refers to a ^∗^*p*-value < 0.05 for 0.1 μg ml^-1^, and a ^∗∗∗∗^*p*-value < 0.0001 for 0.4 μg ml^-1^.

The impact of short-term (2.5–7 h) ciprofloxacin exposure on *qnrS* expression was determined by qPCR amplification of *qnrS* cDNA normalized either to the estimated cell abundance (**Figure 3**), or to the measured cDNA concentration in ng/μl^-1^ (**Supplementary Figure S2**). The normalized expression of *qnrS* was significantly higher in cells grown in 0.1 μg ml^-1^ ciprofloxacin in comparison to those grown without ciprofloxacin; and cells grown in 0.4 μg ml^-1^ ciprofloxacin displayed significantly higher normalized *qnrS* expression levels than those grown in 0.1 μg ml^-1^ ciprofloxacin. In contrast, at 1.0 μg ml^-1^ ciprofloxacin, *qnrS* expression levels significantly dropped, returning to basal levels observed without selection stress.

## Discussion

Plasmids harboring genes encoding for resistance to antibiotics not only confer survival to individual host cells, but also may drive the evolution of entire bacterial communities in specific ecosystems, due to their ability to transfer or be transferred and expressed within closely associated organisms (Bennett, 2008; Cytryn, 2013). A myriad of studies have assessed the dynamics of antibiotic resistance-conferring plasmids from clinical environments, but only few have investigated the behavior of ARG-harboring plasmids isolated from non-clinical ecosystems. We hypothesize that municipal WWTPs may be hotspots for the propagation and dissemination of plasmid-mediated quinolone resistance due to high residual concentrations of fluoroquinolones (especially in biosolids) and the high concentration of biofilm-associated bacteria in these ecosystems (Stokes and Gillings, 2011; Kaplan et al., 2013; Rizzo et al., 2013). Henceforth, the global aim of this study was to assess correlations between levels of selective pressure and bacterial fitness in cells harboring *qnr* plasmids isolated from municipal WWTPs. This was accomplished by assessing growth dynamics, gene abundance and *qnr* expression levels in DH10B *E. coli* electrotransformants harboring two previously described *qnr*-encoding plasmids that were isolated from a wastewater treatment facility (Kaplan et al., 2015). Comprehensive understanding of *qnr* plasmid dynamics in anthropogenic “hotspots” such as WWTPs is crucial due to the high abundance of *qnr* genes in these environments and the ubiquitous presence of sub-therapeutic levels of fluoroquinolones in wastewater effluents and biosolids (McClellan and Halden, 2010; Kaplan et al., 2015).

Plasmid maintenance is believed to come with a distinct fitness cost to the host that is associated with added energetic requirements needed for replication, transcription and translation (Turner et al., 2002). However, in the presence of antibiotics, plasmids harboring ARGs provide a selective advantage to these cells, and therefore maintenance of these plasmids may occur in environments containing residual concentrations of antibiotics. The naïve, plasmid-free *E. coli* DH10B strain, failed to grow even at 0.05 μg ml^-1^ ciprofloxacin, and previously we found that contrary to commonly documented clinical MIC levels for *E. coli* (1 μg ml^-1^) it’s growth was inhibited in ciprofloxacin concentrations below 0.002 μg ml^-1^ (Kaplan et al., 2015). In contrast, the *qnr* plasmid transformants grew well at ciprofloxacin concentrations as high as 0.5 μg ml^-1^. This has significant environmental ramifications since sub-therapeutic concentrations reaching 0.05 μg ml^-1^ have been documented in wastewater effluents and biosolids (Golet et al., 2002; McClellan and Halden, 2010). These products are often used for irrigation or applied as fertilizers in agriculture and therefore may facilitate the dissemination of antibiotic resistance in food webs.

In the complete absence of selection, we found a significant reduction in growth rate and maximal cell density differences between the naïve *E. coli* DH10B cells and the *qnrS* and *qnrB* plasmid electrotransformants. While plasmid maintenance is known to hamper bacterial growth, we believe that this finding also stems from the fact that there is a tradeoff associated with Qnr protection that is caused by competitive binding of Qnr to the gyrase. Although this mechanism lowers gyrase inhibition by fluoroquinolones in a concentration-dependent manner, it also reduces the amount of holoenzyme-DNA, and thereby constrains bacterial growth (Tran et al., 2005).

Although both the *qnrB*- and *qnrS*-harboring plasmids reduced the fitness of host cells in the absence of ciprofloxacin, no significant difference in growth kinetics were observed in the 8 Kbp *qnrS* vs. the 185 Kbp *qnrB* plasmid transformants, despite significant difference in their size. Support for the fact that plasmid size does not significantly impact bacterial fitness was previously demonstrated in *Pseudomonas aeruginosa* (San Millan et al., 2014).

Increased ciprofloxacin concentrations of up to 0.5 μg ml^-1^ resulted in reduced cell density and increased duration of lag phase. However, when ciprofloxacin exceeded 1.0 μg ml^-1^, lag phase was extremely prolonged, after which cell growth did occur. This elongated lag phase may indicate the actual time required for the bacterial cell to transcribe and to translate enough QnrS proteins, to substantially protect the bacterial gyrase, by competing against the antibiotic on binding to its target site.

Another explanation for acquisition of elevated ciprofloxacin resistance in the environment are spontaneous genomic point mutations occurring in the bacterial gyrase/topoisomerase IV genes, as previously demonstrated by Baym et al. (2016), who showed the progression of *E. coli* growth along a ciprofloxacin gradient as a function of well synchronized mutations in various gyrase loci. The occurrence of these mutations may be facilitated by the previously mentioned “window of opportunity” conferred by plasmid-mediated quinolone resistance (Drlica, 2003; Cantón and Morosini, 2011; Marti et al., 2016). Another, more recent study eloquently demonstrated that tolerance boosts the chances for antibiotic resistance mutations to spread within a bacterial population under selective pressure (Paiva et al., 2017). Henceforth, it may be hypothesized that *qnr-*bearing plasmids may increase the capacity of bacteria to acquire bacterial gyrase/topoisomerase IV point mutations).

By applying a qPCR-based approach, we determined that the *qnr*S-bearing plasmid is a low copy number plasmid, averaging 2–3 copies per-cell and that short-term exposure to selective ciprofloxacin concentrations did not affect plasmid copy number. Conversely, this short-term exposure to ciprofloxacin stimulated *qnrS* transcription levels up to fivefold higher than in comparison to cells grown without ciprofloxacin. Elevation in *qnr* expression was previously demonstrated for *qnrB* in response to ciprofloxacin in the growth medium, and this phenomenon was found to be regulated by the cellular SOS-response (Wang et al., 2009). Nonetheless, SOS-independent elevation in *qnrS* levels was found by Rutgersson et al. (2014) who demonstrated that elevated levels of fluoroquinolones in an Indian river sediment coincided with higher expression levels of *qnr* genes, and also by Okumura et al. (2011) who demonstrated induction of *qnrS* transcripts in an SOS-independent manner in *E. coli*. Another study recently applied full transcriptome sequencing to demonstrate that sub-MIC levels of ciprofloxacin changed the transcription levels of several plasmid-borne genes (some of which were resistance genes) in plasmid donor *E. coli* strain in an SOS-independent manner (Shun-Mei et al., 2018). This study also demonstrated that sub-MIC concentrations of ciprofloxacin stimulate inter-species conjugative transfer of plasmids.

As depicted in **Figure 3** and **Supplementary Figure S2**, in contrast to the significant elevation in relative *qnrS* gene expression when exposed to 0.4 μg ml^-1^ ciprofloxacin, under higher ciprofloxacin concentration (of 1.0 μg ml^-1^) no such effect was detected. This may result from the fact that under higher antibiotic concentrations, the competitive binding of the Qnr to the bacterial gyrase fails to sufficiently defend the replication fork. It is possible that under these high ciprofloxacin levels, the cell slows *qnr* transcription and adopts other means of defense, but additional experiments coupled to whole genome sequencing are required to validate this hypothesis.

## Conclusion

This study revealed two important insights. First, it demonstrates that *qnr* plasmids isolated from WWTPs confer a selective advantage in the presence of residual fluoroquinolone concentrations that are often present in WWTPs. This has significant ramifications for understanding the distribution and potential dissemination of these plasmids in irrigated and fertilized environments that receive wastewater effluents and biosolids, respectively. Second, it suggests that under sub-MIC fluoroquinolone concentrations, the protective nature of *qnr* is dependent not on plasmid copy number, but rather on expression level; and that this expression is dictated by fluoroquinolone concentration. This finding is supported by Jiang and colleagues, who showed dependence of the expression levels of *qnrS* on the presence of ciprofloxacin (0.25 μg ml^-1^) in clinical *Enterobacteriaceae* isolates (Jiang et al., 2014). Interestingly, both large and small *qnr-*harboring plasmids conferred selective advantages in the presence of sub-MIC ciprofloxacin concentrations, suggesting that plasmid size does not substantially affect the fitness of host cells. Future studies should focus on elucidating fluoroquinolone concentration-dependent molecular regulatory mechanisms associated with varying level of selection stress and *qnr* expression.

## Author Contributions

EK designed the experiments, conducted the research, and wrote the manuscript. RM performed the real-time PCR experiments, data analysis, and contributed to writing the manuscript. EJ helped to edit the manuscript and EC assisted in experimental design and edited the manuscript.

## Disclaimer

The content of this article reflects only the authors’ views and the Research Executive Agency is not responsible for any use that may be made of the information it contains.

## Conflict of Interest Statement

The authors declare that the research was conducted in the absence of any commercial or financial relationships that could be construed as a potential conflict of interest.
