# Supplementary material for: Enhanced Bacterial Fitness Under Residual Fluoroquinolone Concentrations Is Associated With Increased Gene Expression in Wastewater-Derived qnr Plasmid-Harboring Strains
Source: Front Microbiol. 2018 Jun 8;9:1176. doi: 10.3389/fmicb.2018.01176 (PMC6003256; doi:10.3389/fmicb.2018.01176)
Supplement: Supplementary file 1 [file Data_Sheet_1.docx]

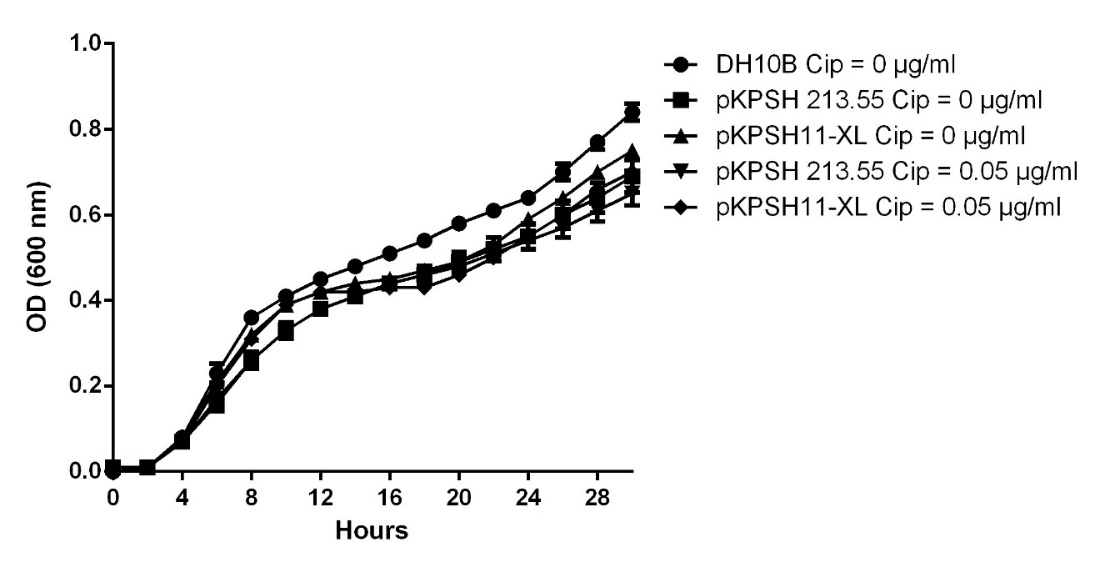


Supplementary Fig. 1: Comparison of growth dynamics of both the naïve DH10B *E. coli* and the electrotransformants DH10B cells carrying either pKPSH213.55 or pKPSH11-XL plasmids. Strains growth was monitored under no selection and under lowest levels of ciprofloxacin (0.05 µg ml^-1^).


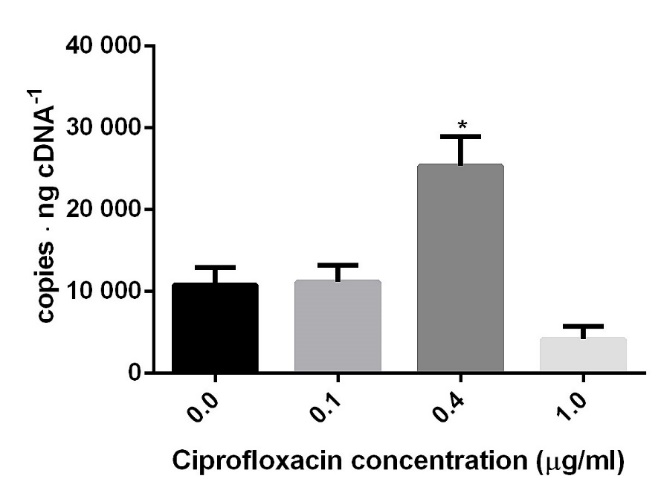


Supplementary Fig. 2: *qnrS* gene expression levels normalized to ng of cDNA at mid-log phase under different ciprofloxacin concentrations (0 µg ml^-1^ , 0.1 µg ml^-1^ , 0.4 µg ml^-1^ and 1.0 µg ml^-1^). The reported significance refers to a p value <0.05.
